# Deriving site-specific clean-up criteria to protect ecological receptors (plants and soil invertebrates) exposed to metal or metalloid soil contaminants via the direct contact exposure pathway

**DOI:** 10.1002/ieam.1528

**Published:** 2014-01-01

**Authors:** Ron Checkai, Eric Van Genderen, José Paulo Sousa, Gladys Stephenson, Erik Smolders

**Affiliations:** † US Army Edgewood Chemical Biological Center, Environmental Toxicology BranchAberdeen Proving Ground, Maryland, USA; ‡ International Zinc AssociationDurham, North Carolina, USA; § IMAR-CMA, Department of Life Sciences, University of CoimbraCoimbra, Portugal; || Stantec ConsultingGuelph, Ontario, Canada; # Division Soil and Water Management, Katholieke UniversiteitLeuven, Belgium

**Keywords:** Bioavailability, Metal, Metalloid, Soil Remediation

## Abstract

Soil contaminant concentration limits for the protection of terrestrial plants and soil invertebrates are commonly based on thresholds derived using data from laboratory ecotoxicity tests. A comprehensive assessment has been made for the derivation of ecological soil screening levels (Eco-SSL) in the United States; however, these limits are conservative because of their focus on high bioavailability scenarios. Here, we explain and evaluate approaches to soil limit derivation taken by 4 jurisdictions, 2 of which allow for correction of data for factors affecting bioavailability among soils, and between spiked and field-contaminated soils (Registration Evaluation Authorisation and Restriction of Chemicals [REACH] Regulation, European Union [EU], and the National Environment Protection Council [NEPC], Australia). Scientifically advanced features from these methods have been integrated into a newly developed method for deriving soil clean-up values (SCVs) within the context of site-specific baseline ecological risk assessment. Resulting site-specific SCVs that account for bioavailability may permit a greater residual concentration in soil when compared to generic screening limit concentrations (e.g., Eco-SSL), while still affording acceptable protection. Two choices for selecting the level of protection are compared (i.e., allowing higher effect levels per species, or allowing a higher percentile of species that are potentially unprotected). Implementation of this new method is presented for the jurisdiction of the United States, with a focus on metal and metalloid contaminants; however, the new method can be used in any jurisdiction. A case study for molybdate shows the large effect of bioavailability corrections and smaller effects of protection level choices when deriving SCVs. *Integr Environ Assess Manag* 2014;10:346–357.

## EDITOR'S NOTE:

This paper represents 1 of 6 articles generated from a workshop entitled “Ecological soil levels: next steps in the development of metal clean-up values” (September 2012, Sundance, Utah, USA). The purpose of the workshop was to provide managers and decision makers of contaminated sites in North America with appropriate methods for developing soil clean-up values that are protective of ecological resources. The workshop focused on metals and other inorganic contaminants because of their ubiquity at contaminated sites and because their natural occurrence makes it difficult to determine adverse levels.

## INTRODUCTION

The guidance for developing ecological soil screening levels (Eco-SSL) in the United States to protect plants, soil invertebrates, mammals, and birds has been available for nearly a decade (USEPA 2002, 2005). The guidelines were derived using generic methods that could be applied to sites within a specific ecological risk assessment (ERA) framework. However, practical application of the ERA paradigm for specific contaminated sites has often suffered during the transition from highly conservative screening level ERA (SLERA) to the site-specific baseline ERA (BERA) that follows. The inappropriate application of conservative soil screening values (e.g., Eco-SSL) as clean-up targets has prompted the need for development of a method for deriving soil clean-up values (SCVs) within the context of the site-specific BERA. The current study provides an overview of existing international approaches for developing soil guidelines and presents a proposed new method for deriving site-specific soil management levels (i.e., SCVs) for plants and soil invertebrates that recognizes site-specific differences in factors that affect bioavailability and the need for flexibility in management requirements for ecosystem protection. Implementation of this new method is presented for the jurisdiction of the United States, although this new method can be used in any jurisdiction. Additionally this method may be used, or modified for its use, with other classes of chemical contaminants if applied within an appropriate context that accounts for other chemical class considerations. Metals and metalloids were selected as the focal point for introducing this method because they are naturally occurring, globally ubiquitous, and extensive data are available in the existing literature. Related articles within the special series (this issue) will focus on fate, bioavailability, and modeling of contaminant metals, wildlife toxicity reference values, food web and wildlife exposure issues, soil microbial processes, and regulatory issues.

**Figure 1:**
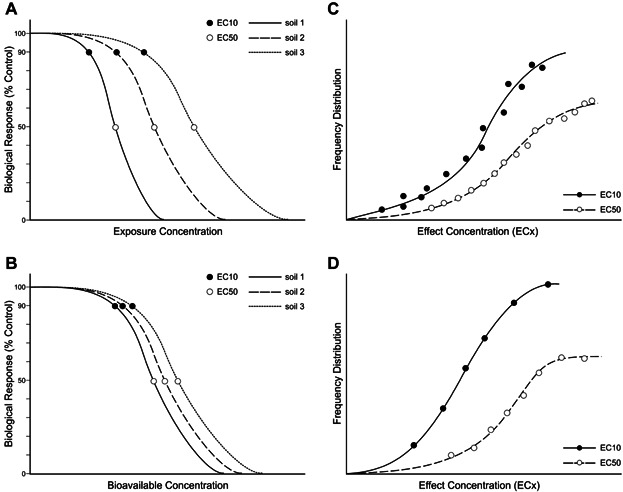
Conceptual scheme for deriving soil limits from spiked soils in different jurisdictions. (A) Biological responses of a species in 3 different soils amended with metal. The toxicity endpoints, here EC10 and EC50, are shown. The endpoint values either vary because soil properties differ, or because the metal has weathered and aged for different lengths of time in the respective soils. (B) Illustration of the same responses but expressed as exposures to bioavailable concentrations (i.e., taking the difference in bioavailability into account by soil extractions, modeling, and normalization). (C) The cumulative frequency distribution of toxicity endpoints (EC10 or EC50 in a toxicity database of a metal, representing different species and soils; soil limits are derived from that distribution at a given percentile of the data [see text]). (D) The cumulative frequency distribution of the same data averaged by species and normalized for bioavailability (i.e., data are normalized on the basis of pertinent reference soil properties).

Soil quality guidelines to protect soil organisms exist for different jurisdictions, including Australia (Soil Ecological Investigation Limit [Soil EIL]) (NEPC 2011a), Canada (Provisional Soil Quality Guidelines [SQG]) (CCME 2006), the European Union (EU) (predicted no effect concentration-soil [PNEC_soil_]) (ECHA 2008b), and the United States (Eco-SSL) (USEPA 2005). Conceptually, all 4 approaches follow similar processes for deriving guidelines specific to protecting soil invertebrates and plants. Data selection and screening are compulsory in any approach, and compilation of ecotoxicological data yields effect concentrations for numerous species. Effect concentrations used for the derivation of limits include no observed effect concentrations (NOEC, highest concentration tested with no significant effect), lowest observed effect concentrations (LOEC, lowest observed concentration that causes a significant effect), and ECx (exposure concentrations yielding a specified percentage effect; e.g., EC50). Jurisdictions differ in the type of endpoints selected but typically select only one endpoint for each test, often the most sensitive one with known ecological relevance. NOEC and EC10 values have been used interchangeably in limit setting in the EU and elsewhere, and it is beyond the scope of this article to make a recommendation regarding whether it is internationally or scientifically defensible to completely eliminate NOEC thresholds from use in deriving soil limit values. Effect concentrations of metals in soils not only differ among different plant and invertebrate species but also among different soils (Roembke et al. 2006; Rooney et al. 2006; Van Gestel et al. 2011). Comparison of response data for tests conducted with the same species illustrates variability in response among soils (Figure 1A), ostensibly related to the difference in bioavailability of metals when added to different soils. To generate generic soil screening levels, as prescribed within Canada (SQG) and the United States (Eco-SSL) guidelines, all effect concentrations from available soil invertebrate and plant studies that meet both acceptance and data quality criteria are used. In Canada, these are also used to plot cumulative frequency distributions of effect concentrations (e.g., EC10, EC50; Figure 1C) on which basis soil limits are derived. However, the prescribed guidelines from Australia (Soil EIL) and the EU (PNEC_soil_) have advanced the approach to include procedures for normalization of the ecotoxicity data based on factors affecting bioavailability. This bioavailability normalization reduces the variation of effect concentrations among soils (Figure 1B). This process collapses all data for a single species and places each into a consistent response curve that can be used to estimate valid species-specific effect concentrations for a given metal. As a final step, the effect concentrations for all species can then be collated to construct frequency distributions (Figure 1D) termed the Species Sensitivity Distribution (SSD) (Posthuma et al. 2001; http://www.epa.gov/caddis/da_advanced_2.html, accessed 23 August 2013). A specific percentile is then selected for the derivation of the limit, the hazardous concentration for y% of the species (HCy), also known as the concentration protecting “100 − y” percent of the species. In addition, before construction of the SSD, data normalization for different soils can be carried out to correct for differences that affect bioavailability, which then allows the derivation of site-specific SCVs. The section below reviews which steps are included in the derivation of soil quality criteria within 4 major jurisdictions (Table1).

**Table 1:** Overview of critical factors within derivation processes for soil screening levels in EU, Canada, the United States, and Australia

	REACH (EU)	CCME (Canada)	Eco-SSL (United States)	NEPC (Australia)
Name of limit	PNEC_soil_, predicted no effect concentration in soil	SQG, soil quality guideline	Eco-SSL, ecological soil screening level	EIL, ecological investigation level
Protection goals	Plants, invertebrates, soil microbial processes	Plants, invertebrates, soil microbial processes	Plants, invertebrates	Plants, invertebrates, soil microbial processes
Data selection				
Substrate	Natural (EU relevant) or OECD soil	Natural or artificial soil	Natural (preferred); USEPA/ASTM/OECD standard artificial soil	Natural (Australian; EU/OECD; Australia relevant soils)
Background information	Soil properties required for bioavailability corrections	Soil properties required to establish implicitly high bioavailability scenarios for inorganic substances	Soil properties collected (pH and OM%); matrix determines soils with properties for high relative bioavailability	Soil properties required for bioavailability corrections
Test duration	Chronic	Chronic preferred; short-term durations not excluded	Chronic	Chronic
Biological endpoints (direct effects)	Growth, reproduction, survival	Growth, reproduction, survival, mortality, behavior, lesions, physiological changes, respiration, nutrient cycling and/or decomposition, genetic adaptation, visible injury to plants; uncertainty factors for short-term data	Plants (by preference): growth (biomass), physiological parameters; invertebrates (by preference): reproduction, population parameters, growth	Growth, reproduction, survival
Substance	Single metal salt	Single contaminant	Single metal salt	Single metal salt
Correction for bioavailability				
Normalization for soil properties	Yes	No	No	Yes
Correction for aging and salt effects	Yes	No	No	Yes
Derivation of limit				
Endpoints utilized	NOEC or EC10, corrected for bioavailability	Order of preference: EC/IC25 (± 5); LOECs/NOECs and EC50s; LOECs with or without UF; EC/LC/IC50 with or without UF	Order of preference: EC20, MATC (geomean of NOAEL and LOAEL), EC10	NOEC or EC10, LOEC or EC30, EC50; all corrected for bioavailability
Derivation of limit from endpoints	Geomean of species NOEC or EC10; HC5 derived from 5th percentile of SSD; PNEC_soil_ = HC5/AF, where typically the AF = 1 to 5	TEC = 25th percentile of SSD of the EC/IC20–30s or specified combination of LOECs/NOECs/EC50s for residential, agricultural, and/or park land uses; TEC = 50th percentile for industrial and/or commercial lands; TEC = lowest LOEC/UF for residential, agricultural, and/or park-lands, or lowest LOEC for commercial and/or industrial; TEC = lowest EC50 or LC50/UF for all land use classes where UF = 5 or 10	Eco-SSL is geomean of benchmarks (above), using those (3 minimum) from soils that scored higher for bioavailability (on a defined bioavailability matrix of relevant soil properties)	Ecological Investigation Level, EIL (SSD of geomeans of species LOEC or EC30, at protective concentration corresponding to HC1, HC20, or HC40, respectively, for pristine environments, urban residential and/or open space, or commercial and/or industrial areas)

AF = assessment factor; EU = European Union; UF = uncertainty factor; TEC = threshold effect concentration.

## OVERVIEW OF INTERNATIONAL APPROACHES TO DERIVING SOIL QUALITY CRITERIA

### United States

Eco-SSL were developed as screening tools to be used within SLERA, specifically in Step 2 of the Superfund (i.e., Comprehensive Environmental Response, Compensation, and Liability Act [CERCLA]) ERA process. The US Environmental Protection Agency (USEPA) developed Eco-SSLs to conserve resources by limiting the need for the USEPA and other risk assessors to perform repetitious toxicity data literature searches and data evaluations for the same contaminants for every ERA Superfund site. Proper application of Eco-SSL values provides information about contaminants and soils that warrant further consideration in the site-specific BERA but also delineates those contaminants that may be excluded from further investigation.

For plant and soil invertebrate species, the corresponding Eco-SSLs include concentrations of contaminants in soil that are considered protective of these receptors, because they were derived with a preference for conditions of high bioavailability thereby providing a conservative screening tool. The major exposure pathways considered for organisms intimately associated with soil are direct contact with soil (plants and soil invertebrates) and soil ingestion (soil invertebrates) (USEPA 2005). The Eco-SSL methods also considered birds and mammals exposed to soil via the food chain; however, those procedures and corresponding screening levels are discussed in a companion article (Mayfield et al. this issue).

Derivation of Eco-SSLs for plants and soil invertebrates follows a series of steps (Table1), including a detailed scrutiny of available literature, selection of relevant articles that satisfy rigorous acceptability criteria to ensure data quality, followed by the sorting of studies on the bases of completeness, rigor of testing, and bioavailability criteria. Currently, Eco-SSL values are available for 17 metals and/or metalloids, and 4 types of organic compounds. The Eco-SSL for each chemical is not a percentile of an SSD but is calculated as the geometric mean of effect concentrations of the subgroup of data with highest relative bioavailability (USEPA 2005). The effect concentrations used in Eco-SSL derivations are (in order of preference): EC20, MATC (the geometric mean of NOAEC and LOAEC; where the NOAEC value is the highest concentration tested that produced no observed adverse effect, and the LOAEC value is the lowest observed adverse effect concentration reported), or EC10. In this respect, the Eco-SSL does not follow the paths of Figures 1A, 1C, and 1D to derive limits, but includes considerations of factors affecting bioavailability in a matrix approach. Development of Eco-SSLs includes data for species from different taxonomic groups, representing a range of sensitivities from the spectrum of diverse ecological functions attributed to organisms comprising soil communities: primary producers and different functional groups of soil invertebrates. Eco-SSL assumptions used within their derivation specify use of the most sensitive endpoints under high bioavailability scenarios, thus misapplication of Eco-SSLs as SCVs can lead to needless costly soil remediation.

The major limitations of the Eco-SSL approach are that Eco-SSLs for terrestrial plants and soil invertebrates are appropriate only to aerobic soils where key soil parameters fall within a specific range (4 ≤ pH ≤ 8.5 and organic matter content ≤10%). The Eco-SSL process for plants and soil invertebrates takes into account contaminant bioavailability by prioritizing data inclusion, rather than using data normalization and an SSD approach. Furthermore, at the time the Eco-SSL process was developed, the USEPA considered that microbial data were insufficient and interpretation of test results too uncertain to establish risk-based thresholds, thus are not included in Eco-SSL derivation (USEPA 2005). Eco-SSLs for terrestrial plants and soil invertebrates were intentionally designed as conservative generic screening values, not to be used as clean-up standards, therefore USEPA emphasizes that it is inappropriate to adopt or modify Eco-SSLs as SCVs.

### Canada

In Canada, metals are regulated on the basis of a 3-tiered risk-based framework (CCME 2006). Tier 1 comprises SQGs (formerly called criteria) that are considered to be relatively conservative and protective of most ecological receptors. Benchmarks are derived to protect both human health and ecological receptors, for multiple exposure pathways, different soil textures, surface and subsurface soils, different land-use classes, and in consideration of proximity of surface and groundwaters (CCME 2007). In total, Canadian SQGs are derived for 5 exposure pathways, and the SQG for protection of the environment (SQG_E_) is derived using some combination of these values, depending on the land use classification (Supplemental Data Figure 1). For an ecological risk assessment of a site, the risk associated with the lowest Tier 1 SQG for a metal (i.e., the final soil quality guideline [SQG_F_]) and a specific land-use class can potentially trigger further investigations at subsequent tiers if the SQG_F_ value is exceeded. Inherent to the process is the understanding that the level of protection is more stringent for land classified as agricultural, residential, and park lands compared to those classified as commercial or industrial (Table1).

The process used to derive the soil standards or guidelines depends on the regulatory jurisdiction within Canada. For lands that fall under the federal jurisdiction, the derivation protocol that applies was developed by the Canadian Council of Ministers of the Environment (CCME 2007). For lands that fall under provincial jurisdictions, the provincial governments have either adopted the federal protocol or have developed a provincial process that is, in principle, similar to the federal protocol. For example, the provinces of British Columbia, Alberta, and Ontario have protocols for deriving screening values that are unique to their jurisdictions (BC-MOE 2006; Alberta Environment 2010; Ontario-MOE 2011).

Canadian guidelines for the direct contact exposure pathway (SQG_SC_) are derived using 3 methods: 1) weight-of-evidence, 2) LOEC, and 3) median effects (Supplemental Data Figure 2). The method used depends on the nature of the quality and quantity of the data available from which to make a toxicological assessment. The weight-of-evidence method (Supplemental Data Figure 2) is implemented when data sets are rich in both quantity and quality, thus allowing the identification of either a threshold effect concentration (TEC) for agricultural, residential, and/or parkland uses, or an effects concentration-low (ECL) for commercial/industrial land uses. SQG_SC_ can be derived from an SSD of ranked values (i.e., IC25/EC25 values ± 5%; where ICx is the concentration that yields x-percentage inhibitory effect), and a specified percentile for protection (i.e., protection level). The minimum data requirement for this method is at least 10 data points from at least 3 studies, with representation of a minimum of 2 soil invertebrate and 2 plant species. When minimum data requirements are met using a combination of effects data (e.g., EC50, LOEC, NOEC), a frequency distribution using an empirical distribution function with averaging can be used, as long as there is the absence of statistical bias in the data. For redundant toxicity estimates (endpoints and/or species), the geometric mean of the acceptable values is used. The 25th percentile of the distribution of ranked values is used as the basis for the soil contact guidelines for agricultural, residential, and/or park lands, and the 50th percentile for commercial/industrial lands. The derived TEC might be divided by an uncertainty factor (UF) between 1 and 5, depending on the magnitude of the uncertainty; guidance is provided but application of a UF relies on best professional judgment of risk managers.

When data are limited (i.e., weight-of-evidence method requirements are not met), the TEC is derived by dividing the lowest LOEC by an uncertainty factor (≥1) for agricultural, residential, and/or park land uses and the ECL is derived using the geometric mean of the available LOEC data. This approach constitutes the LOEC method (Supplemental Data Figure 2). A minimum of 3 studies reporting LOEC endpoints are required, and at least one terrestrial plant and one soil invertebrate species must be represented. The UF can range between 1 and 5, with criteria provided in the guidance for defining the uncertainty. The median effects method (Supplemental Data Figure 2) is used when minimum data requirements cannot be met for either the weight-of-evidence or LOEC methods. The TEC is determined by dividing the lowest EC50 or LC50 by a UF of either 5 or 10, respectively.

Deriving an SQG involves an extensive literature search of all available published and nonproprietary data. Literature data relevant to the direct soil contact exposure pathway are screened to ensure that studies provide scientifically verified information that were developed under conditions of optimal bioavailability. Acceptability criteria for data quality and quantity have been established for the soil-contact derivation process and data must meet minimum requirements for 1 of the 3 different methods described above. In the absence of data, a provisional guideline can be established by adopting a value derived by other agencies.

The strength of the Canadian approach for establishing SQGs is that the CCME process is transparent, in that the rationale and data for each substance (e.g., a metal) are summarized in technical supporting documents. Land use classes and the process for data selection and quality are clearly defined; the derivation process allows for 3 methods to derive SQGs, depending on the quantity and quality of data. There is a provision for derivation of SQGs to protect nutrient and energy cycling (SQGNEC) with a “check mechanism,” when data are limited that allows inclusion of decomposition and respiration data if these data meet quality objectives, and, there is an offsite migration check for commercial and industrial lands. Once the SQG_F_ is established (the final SQG; the lower value of the SQG_E_ and the SQG for Human Health [SQG_HH_]), there are provisions for implementing management and nontoxicological considerations, incorporating plant nutrient requirements, and addressing background concentrations.

Limitations to CCME methods for deriving SQGs are that UF values are used to account for data limitations, and their application is based on professional judgment, without a scientific basis. Toxicological measurement endpoints are diverse with questionable relative ecological relevance, and, bioavailability is not explicitly taken into consideration. No consideration is given to the presence of other metals as cocontaminants (e.g., metal mixtures). If the SQG_F_ value is below geochemical background concentrations, then the geochemical background becomes the final SQG_F_. If the SQG_F_ is below the practical quantitation limit, then a caveat must be added but no adjustment is made. The process for deriving SQG in Canada is comprehensive but complex, and results in a high degree of protection. As a result, the Tier 1 SQG values are used more for screening than to inform for soil remediation.

### European Union–Registration Evaluation Authorisation and Restriction of Chemicals

In the EU, soil metal limits have been developed for the Registration Evaluation Authorisation and Restriction of Chemicals (REACH) regulation regarding the management of chemicals. The limits, PNEC_soil_, are used to identify if current release of metals to soil poses a risk for soil organisms (i.e., plants, invertebrates, and microbial processes). Effects on wildlife are dealt with in a different assessment, and the term PNEC_soil_ is only applicable to plants, invertebrates, and soil microbial processes. The PNEC_soil_ values might be considered equivalent to screening values in other jurisdictions (Table1); they are not SCVs. Several metals have already been evaluated under REACH or were developed under the Existing Substance Regulations that preceded REACH. Derivation of the PNEC_soil_ for individual metals is based on the results of laboratory toxicity tests, almost invariably with natural or Organisation for Economic Co-operation and Development (OECD) artificial soils into which the metal is amended in a soluble form, then fully mixed into the test soil. Toxicity data from tests with metal mixtures are not acceptable for derivation of PNEC_soil_ values, because the measured biological responses must be attributable to the chemical being registered; the exception are field data used on a case-by-case basis in the deliberation of assessment factors (AF; arbitrary values to enhance conservatism) that can be applied in deriving the PNEC_soil_. Data from chronic tests are acceptable, and endpoints for plants and invertebrates should be the most sensitive among the ecologically relevant ones such as growth, reproduction, and survival of plants and invertebrates. Soil properties that are required for bioavailability corrections should be measured and reported (e.g., soil pH, organic matter content, cation exchange capacity, particle size distribution, etc.). NOEC or EC10 data from chronic exposure studies, corrected on the basis of the major soil factors affecting bioavailability, are used to calculate the PNEC_soil._ Details of bioavailability correction are described elsewhere (Smolders et al. 2009) and are also adopted in the REACH guidelines for risk assessments specific for metals (ECHA 2008a). The procedures are based on a large experimental program that led to models for bioavailability. In short, NOEC or EC10 values, expressed as added metal concentrations, are multiplied by a leaching–aging factor (LAF; values ≥1) to account for the lack of full equilibration of the metal in the toxicity tests. This factor is derived from experimental comparisons of differences in toxicity between long-term aged and freshly spiked soils. It accounts for reduced toxicity that is attributable to the removal of the influence of using metal salts (salinity stress, acidification), which diminishes on natural leaching of salts from soils over time. This LAF factor is only applied to the added metal in short-term tests and not to the background or ambient concentration that is naturally aged, nor to long-term tests (>120 days). Next, thresholds are normalized for differences in metal availability between the tested soil and the soil (e.g., reference soil) for which the PNEC_soil_ is calculated, using the slopes of log–log regression lines between toxicity endpoints (e.g., EC50) and the soil abiotic factors (e.g., the effective cation exchange capacity [eCEC]). The formal expression for correction is(1)
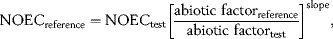
where *reference* is the soil for which the PNEC_soil_ must be derived, *test* is the tested soil, and *abiotic factor* is the soil property with which toxicity is correlated (e.g., eCEC). If the model describing the relationship between the biological response and the exposure concentration has more than 1 soil abiotic factor as an explanatory parameter (e.g., eCEC and %OM), the equation (Eqn. 1) is extended with a product of the 2 ratios. The geometric mean of bioavailability-corrected NOEC or EC10 values are calculated per species, an SSD is plotted, and the 5th percentile of a log-normal distribution calculated (HC5) that protects 95% of the species (Aldenberg and Slob 1993). Finally, PNEC_soil_ is derived as the HC5/AF, where the AF can range between 1 and 5, depending on data richness, validation with field data, etc. For example, AFs were 1 for Cu, and 2 for Ni, in the determination of their respective PNEC_soil_ values (Smolders et al. 2009).

One of the strengths of the REACH program is that a method was developed for the incorporation of bioavailability. Bioavailability corrections were only available for a number of metals, whereas for other metals, such as Cd, no corrections were made and a single, generic, HC5 can be derived. User guidance for the bioavailability calculation, and the calculator, are available to the public in spreadsheet form (Arche 2012). Bioavailability corrections have been used to establish clean-up limits in Belgium by increasing from HC5, to HC25 or HC50, depending on the metal or land use (OVAM 2008).

Limitations to the REACH method are that the PNEC neither accounts for land use, nor sets PNEC_soil_ values at different levels of protection. With respect to site-specific clean-up values, limitations include that the PNEC_soil_ is still based on metal–salt spiked soils, hence no consideration is given to the effect of the source of the metal. In addition, effects of metal mixtures are not accounted for by the PNEC_soil_; these limitations also apply to all other jurisdictions discussed. Even when metal salts were allowed to fully equilibrate in soils, this is still different from soils where sparingly soluble metal sources (e.g., mine waste, slag, sewage sludge) are added to soil to determine toxicity.

### Australia

Australia's guidance, “Schedule B5b” (NEPC 2011a), describes an approach for deriving SQG for contaminated soils (Table1). The Australian method was developed to protect soil processes, soil biota (flora and fauna), and terrestrial invertebrates and vertebrates. Three sets of SQGs termed SQG_(NOAEC and EC10)_, SQG_(LOAEC and EC30)_, and SQG_(EC50)_, correspond to the type of ecotoxicity data sets used in their generation (identified in their subscripts, respectively). The companion guidance document “Schedule B5c” (NEPC 2011b) prescribes development of soil ecological investigation levels (EILs). The National Environment Protection Council (NEPC) adopted the SQG_(LOAEC and EC30)_ for derivation of EIL values, with the intention that when these EILs are exceeded that moderately harmful effects might occur. Schedule B5c prescribes EIL values for chemicals that include the metals Cu, Ni, and Zn. Values for Mo are reportedly under development. EILs are derived for 3 land-use classes, on the basis that each group merits different levels of environmental protection: 1) national parks and/or areas with high ecological value, 2) urban residential and/or public open space, and 3) commercial and/or industrial. The level of protection provided depends on land use and whether the contaminant in question biomagnifies. Neither Cu, Ni, or Zn were found to biomagnify appreciably; therefore, only toxicity due to direct exposure is considered. The recommended levels of protection for species when a contaminant does not biomagnify are the 99th percentile for national parks and/or areas with high ecological value, the 80th percentile for urban residential and/or public open space, and the 60th percentile for commercial and/or industrial areas, respectively.

The Australian method for deriving respective EILs for the metals involves several critical steps. Data collection and data quality criteria are relatively similar to those used in the EU under REACH (see above). The toxicity data are expressed on the basis of added concentration of contaminant, and chronic or sensitive life stage toxicity data are considered; exotic species are not included when data are available for ≥4 native species. Bioavailability factors are included in data normalization, as in the EU method, before construction of the SSD. First, the LAF is applied to correct data arising from laboratory-spiked metal toxicity tests where presence of excess salts and short exposure durations occur. The LAFs have been established based on long-term effects in metal spiked soils in field plots (up to 3 years) compared to freshly spiked soils. Second, the toxicity data are normalized to a standard reference soil or to a specific soil onsite using site-specific information (Figure 1B and Eqn. 1). SSDs are constructed (Figure 1D) using the Burr type III SSD method (Shao 2000) and the added contaminant limit (ACL) derived, based on fixed percentiles (HC1 to HC40) as described above. Finally, the EIL is calculated as the sum of ACL and the ambient background concentration for the site soil (i.e., the contaminant concentration from a clean reference site with a comparable soil type to the site being examined).

The strength of the EIL derivation approach is that it is risk-based and enables protection of a selected percentage of species. The EIL accommodates different types of toxicity data, land uses and purposes, incorporates bioavailability considerations, and final values are never lower than ambient background (because background concentration is added at the end); this aspect is in contrast with the EU method. Finally, this method incorporates recent advances in risk assessment, terrestrial toxicity, and soil chemistry.

Most limitations of the EIL derivation approach are similar to those of the EU: it does not account for bioavailability differences arising from different sources, forms, or speciation of contamination. In general, both EU and Australian methods are relatively complex due to comprehensiveness of the methods.

## DESCRIPTION AND RATIONALE FOR THE PROPOSED NEW METHOD FOR DERIVING SOIL CLEAN-UP VALUES

Building on the methods and procedures used in the EU and Australia to derive screening levels for metals, and on the conceptual framework presented in Figure 1, the following subsections present a proposed new method for deriving site-specific SCVs for metals. It is presented in the context of implementation within the United States; however, this method can be applied in a BERA within any jurisdiction. In general, recommendations follow the bioavailability corrections methods developed in Australia, which means that the added risk approach is used (by which all SCVs are above ambient background concentration). A summary of the steps involved in applying the new method for deriving site-specific SCVs to protect the ecological receptors plants and soil invertebrates is presented in Supplemental Data Table 1.

### Data selection: Quality criteria and endpoints

#### Quality criteria

Data selection criteria for deriving SCVs applicable to contaminated sites in the United States should be based, in part, on guidance established for the derivation of Eco-SSLs (USEPA 2005) but updated to include direct use of soil parameters (abiotic factors; Eqn. 1) that influence the biological responses of organisms. Precedents have been established by the European Community (REACH framework; ECB 2008) and the Australian EIL framework (NEPC 2011a). The focus of the data selection must not be constrained by the first Eco-SSL criterion (i.e., selection of studies where “testing was done under conditions of high bioavailability”) and data from studies with a broader range of environmental conditions should be included. For the derivation of SCVs, the recommendation is to address and incorporate adjustments for site-specific bioavailability differences during the BERA process.

Data generated from standardized toxicity tests (e.g., International Organization for Standardization [ISO], Environment Canada [EC], USEPA, or OECD) carried out with natural or artificial soils amended with a single metal are preferred, as long as the appropriate soil properties are reported. Data from nonstandard tests are allowed where the experimental design is described (i.e., appropriate experimental control treatments, number of test concentrations, number of treatment replicates, amount of soil and number of organisms in each test unit), and the source of plant and soil invertebrate test organisms and the methods and procedures are all explicitly documented. The form of the metal tested must be reported, and measured concentrations are preferred over nominal ones. The methods used to derive ECx concentrations and confidence limits should be reported or calculated from reported data, with ECx concentrations reported as the added total-concentrations (i.e., total treatment concentrations corrected for background).

#### Endpoints

Toxicity endpoints estimated from the data generated from tests with prolonged exposures (chronic, definitive, sublethal tests) of organisms to the contaminated soil have higher ecological relevance and are preferred over those from short-term exposures. Data from mesocosms and controlled field studies (e.g., metal-amended field plots) can be used as long as data for single species (population level) permits the calculation of the required effect concentrations.

Measurement endpoints for plant species include biomass (above and below ground), seedling emergence, wet and dry mass metrics, root and shoot elongation, yield, and flower bud production or seed production. Biochemical markers of plant exposure (stress enzymes, chlorophyll content, etc.) are not recommended because their ecological relevance is less clear. The ECx values for plant mass and yield metrics are the most frequently reported toxicity endpoints. Seedling emergence is known to be a relatively insensitive and acute endpoint with respect to metal contamination, and there is inherently greater variability in the data for wet mass metrics than for dry mass. For invertebrates, measurement endpoints include survival, growth (dry mass), or reproduction (e.g., progeny and/or cocoon production). Currently, soil invertebrate endpoint measurements for biomarkers, avoidance behavior responses, or changes in enzyme status should not be included in the data set. Measurement endpoints for microbial processes can be included; however, a discussion on this is beyond the scope of this article (Kuperman et al. this issue). Measurement endpoint data for soil microbial processes are not required for derivation of SCVs protective of plants and soil invertebrates; however, when microbial data are available and included, the resulting SCVs are protective not only of plants and soil invertebrates but are also protective of the associated soil microbial processes.

### Determination of ambient background concentrations

The Australian method for the derivation of EIL values for metals or metalloids requires the discernment of naturally occurring or ambient background concentration and the added contaminant concentration. This is the so-called added risk approach (Struijs et al. 1997; Crommentuijn, Polder et al. 2000; Crommentuijn, Sijm et al. 2000). The added risk approach attributes low-to-nil contribution of the ambient background concentration to toxicity, and ascribes that the ambient background concentration has resulted in biodiversity of the ecosystem or provided the levels of micronutrients (e.g., metals and/or metalloids) necessary for the nutrition of organisms in the environment (Traas 2001). Therefore, the added risk approach only considers the effects of contaminants added to the environment and has been used as a relevant approach for ERA.

Because ambient metal (and metalloid) concentrations differ among soils, such natural variability typically poses challenges for conducting ERA for terrestrial receptors. The USEPA guidance for exposure assessment indicates that assessment of metal levels should be inclusive of background concentrations. Risk assessors evaluating risks at the regional and local (site) scales can comply by accounting for the natural occurrence of metals either at the beginning of an assessment (Problem Formulation), during the BERA, or when making risk management decisions about the implications of the predicted or observed levels of metals in soils (USEPA 2007). In addition at a national level, USEPA also allows discernment of metal concentrations, which include the background concentrations, that are considered protective for wildlife, plants, and soil organisms (USEPA 2005). Thus methods for determining an ACL can be invoked as a next logical step following initial soil screening, as long as its application includes the addition of an appropriate ambient background concentration into the resulting total metal and/or metalloid concentration for assessing a site. When proceeding to the BERA, this added risk approach can also provide critical widely applicable ACL information that, in conjunction with ambient background concentration, moves ERA meaningfully toward determining remediation levels on a site-specific basis. In the United States, this approach has additional utility because, by calculation, these site-specific acceptable concentrations are never below the site-specific background concentrations and, by policy, the USEPA does not require clean-up to levels below site-specific background concentration (USEPA 2002).

As an initial comparison at the beginning of a BERA, a regional or local background concentration can be inserted into this calculation even before a site-specific ambient background concentration is measured (or otherwise established) for the site undergoing assessment. The US Geological Survey (USGS) has carried out national surveys of the ambient background concentrations of metals across the United States, and these data are readily available in publications (Shacklette et al. 1971; Shacklette and Boerngen 1984) as well as online (USGS 2012). Furthermore, other surveys describe relationships between soil concentrations and plant uptake and provide comparable ambient background concentrations (Holmgren et al. 1993; White et al. 1997). Holmgren et al. (1993) correlated their soils information using detailed soil surveys, available from the Natural Resources Conservation Service (NRCS; formerly Soil Conservation Service [SCS]). Detailed soil surveys identify related soil series and soil types by county across the United States and are available online (http://www.nrcs.usda.gov/wps/portal/nrcs/main/national/soils; accessed December 17, 2013) or often in printed form at NRCS county offices. Soil relationships and data given in detailed soil surveys are especially useful for identifying locations to sample in the BERA, to determine ambient background concentration for closely related soils.

### Corrections for bioavailability

There is now sufficient experimental evidence that total metal thresholds vary widely (>factor 10) (Rooney et al. 2006) among soils with different properties and between freshly spiked and corresponding aged or field contaminated soils (factors 1 to approximately 10) (Smolders et al. 2009). Without corrections for bioavailability, derived SCVs for metals are bound to frequently be low and impractical (below natural background of several types of soils), because such values derived without correcting for bioavailability logically protect worst case scenarios. With corrections, SCVs were shown to be above natural background (Smolders et al. 2009), thus allowing for site-specific management.

The models to correct for bioavailability range from mechanistic to empirical. In both the REACH and Australian programs described above, bioavailability normalization was made with empirical toxicity-based models, accounting for major factors affecting bioavailability. These models correct for 2 processes: 1) using a generic- or soil pH-dependent factor on EC10 and/or NOEC data to correct for the alleviation of toxicity upon the aging and removal of confounding factors present in spiked soils, and 2) using Equation 1 for the differences in toxicity among soils. By correcting, the thresholds derived using spiked soils are converted to a value that is specific for a soil type to which one wants to assess the toxicity, and for a soil in which the toxic metal is equilibrated. We recommend following the empirical approach described herein, rather than using mechanistic models (e.g., t-BLM, and electrostatic models; Thakali et al. 2006) because the mechanistic models require that soil speciation codes are included in the calculation and these models have not yet been validated on aged soils, in contrast with the empirical approach (Smolders et al. 2009).

### Determination of species sensitivity distribution

Effect concentrations from toxicity tests, normalized for bioavailability on the basis of factors affecting it, can subsequently be averaged per species to yield a valid species-specific ECx; geometric means (geomeans) are preferred over arithmetic means, as data are often log-normally distributed. SCVs can be calculated at different levels of effect (e.g., EC10, EC20, etc.). The selection of the ECx level is not a scientifically based decision but is a regulatory choice and determines the level of effect on species populations that is acceptable. In general, EC10 values indicate nil-to-marginal effect, and usually fall within the “noise-level” of the control response whereas EC20 values are an index of an incipient detectable effect. The cumulative frequency distribution of geomean values (Figure 1D) often shows log-normal rather than normal distribution. HCy values (i.e., percentiles) derived from the cumulative frequency distribution can be calculated, using standard methods for their derivation (Aldenberg and Slob 1993; Shao 2000). Note that HCy values are often a statistical extrapolation, not interpolation of the data, and that these become increasingly dependent on the assumed curve fitted to the SSD when either y (i.e., y in HCy) is small or the number of species is small. For metals and/or metalloids for which rich toxicity data sets exist (i.e., large number of data within the SSD), the HCy is rather insensitive to the selection of the corresponding curve; however, its choice can be critical for data poor substances. Statistical tests (goodness of fit) may be advocated, although the use of different ECx curves for different metals may result in a situation where not all substances are treated equally; such inconsistency should be considered from a regulatory point of view.

### Use of assessment factors

The use of AFs (also known as uncertainty factors, application factors, or safety factors) has been implemented by several jurisdictions in various ways to account for remaining uncertainty when extrapolating from laboratory toxicity test data to the field. Similarly, AFs have been applied to extrapolate from acute to chronic endpoints, interspecies relationships, and to compensate for deficiencies in the available toxicity data set. Generally, the magnitude of the AF is inversely related to the size of the data set, or the preponderance of certain endpoints (acute or chronic). For example, AFs for deriving the soil limit under the REACH legislation range between 1 and 1000 (ECHA 2008b).

Use of AFs has prompted debate for several reasons. Foremost, the magnitudes of AFs have been arbitrarily developed and applied without a universally accepted method. Existing AF methods do not provide transparency regarding the degree of protection afforded by a certain magnitude and thus do not permit informed decision making. Other criticisms of AF methods include ignoring all other data except the lowest toxicity values, having no theoretical or scientific basis. Furthermore, such a procedure is at odds with a risk-based approach, which requires a compilation of data to derive estimates of the probability of certain ecological risks occurring.

For the purposes of deriving soil clean-up limits, it is assumed that sufficient data exist in the literature to satisfy the requirements for taxonomic diversity (e.g., 6 to 8 taxonomic groups, as discussed in EU), and a robust SSD can be constructed. Given that this is the case for the metals included within the existing Eco-SSL framework for protection of plants and soil invertebrates, AFs should not be applied when deriving SCVs.

## CASE STUDY

**Figure 2:**
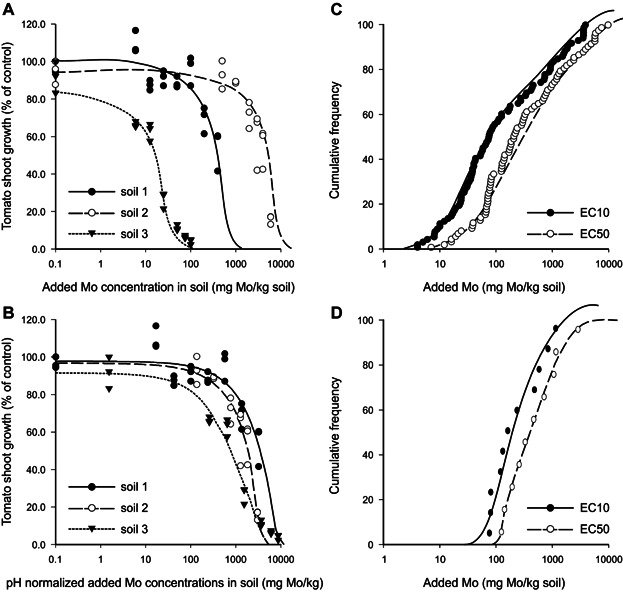
A case study to derive soil clean-up values (SCVs) from toxicity data for Mo (added as molybdate to soil). (A) Tomato shoot growth in 3 different soils amended with Mo, most sensitive soils have highest pH due to lower sorption of the Mo anion (McGrath et al. 2010). (B) Biological data as in (A) but total soil Mo concentrations corrected to that in soil of pH = 6.3 using the empirical relationship between toxicity and pH (McGrath et al. 2010). (C) Frequency distribution of toxicity endpoints (EC10 or EC50) of all Mo data (4 different plants [McGrath et al. 2010], 3 invertebrates [van Gestel et al. 2011], and 3 microbial processes [Smolders and Buekers 2009] tested in 10 different soils). (D) The cumulative frequency distribution of the data averaged by species and normalized for bioavailability to a soil with pH = 6.3 and 10% clay.

Data for the ecotoxicity of the metalloid Mo were analyzed to identify to what extent the bioavailability corrections (normalization), choices of effect levels (ECx), and protection of species (HCy), affect the final SCVs. The data are based on studies recently reported (Smolders et al. 2009; McGrath et al. 2010; van Gestel et al. 2011, 2012). These studies revealed that the relationships for bioavailability with soil clay content and soil pH allowed for normalization of toxicity data, and illustrate that the application of LAF is defensible because of a significant and large proportion of the variance was explained. Figure 2 and Table2 show the influences of bioavailability corrections for 3 soils, choice of SSD (based on ECx), and selection of the level of protection (HCy), on derived ACL values for Mo.

**Table 2:** The soil ACL for Mo as affected by the choices based on selections of the level of protection (HCy) and ECx SSD a

	HC5 mg added Mo/kg soil b	HC25 mg added Mo/kg soil b	HC50 mg added Mo/kg soil b
All data, uncorrected for bioavailability			
EC10	5	31	113
EC50	16		
Data corrected for bioavailability (reference soil, pH 6.0, 10% clay)			
EC10	34	130	328
EC50	84		
Data corrected for bioavailability (sensitive soil, pH 7.0, 10% clay)			
EC10	10	59	206
EC10	21		

ACL = added contaminant limit; SCVs = soil clean-up values.

a These choices directly affect derivation of site-specific SCVs, when HCy is normalized to site-specific soil characteristics and subsequently added to site background concentration. The HCy refers to the yth percentile of the cumulative frequency distribution (Figure 4C) of the ECx values, the latter refers to the x% effect threshold on a population in an exposure-response curve (Figure 2D). ACL values derived from data for 4 different plants (McGrath et al. 2010), 3 invertebrates (van Gestel et al. 2011), and 3 microbial processes (Smolders et al. 2009).

b Background not included.

First, bioavailability corrections largely affect the response curves. Figure 2A shows contrasting effects of Mo in 3 soils with pH between 4.4 and 7.8. The toxicity of the added molybdate anion largely increases with increasing soil pH because anion sorption decreases with increasing pH (McGrath et al. 2010). Using the empirical regression between toxicity and pH, and using Equation 1, soil Mo was normalized to a reference soil pH = 6.3 (Figure 2B). This transformation of total soil Mo greatly reduces differences among soils, hence the curve in Figure 2B refers to the toxicity expected in soil with pH 6.3. The SSD of toxicity data for plants, invertebrates, and soil microbial processes are plotted in Figure 2C, either for EC10 or EC50 values (i.e., added Mo concentration basis). By normalizing the data for bioavailability, including corrections for aging and weathering, and by calculating geomean values for each species, the distribution becomes markedly steeper with much lower variance of the data; this result is shown in Figure 2D. Without bioavailability corrections it is not recommended that data be averaged by species, because generic limits derived from that approach would protect soils with average bioavailability but not the wide range of soils encountered in the environment.

The case study illustrates that several choices can be made to derive SCVs from SSDs (i.e., selection and combinations of ECx and HCy). Different combinations can now be examined for the derivation of ACLs, and subsequent calculation of site-specific SCVs (e.g., HC5 of the EC50 values, or HC50 of EC10 values). In this Case Study, the EC50 values in the SSD are not far from the values in the EC10 SSD, and the 5th percentile of the EC50 values (i.e., 95% of the species protected from 50% effect on population) yields lower ACLs than that of the 50th percentile of EC10 values (i.e., 50% of the species protected from 10% effect on population) which corresponds to higher ACLs (Table2). There is no proof yet regarding which of these combinations most likely indicates incipient toxicity in the field; the former may be an index of substantial stress (EC50) on a few number of species (5th percentile), whereas the latter is an index of relatively weak stress (EC10) on a larger number (50th percentile) of species.

Mo concentrations in US soils have been reported in the range of <3 to 15 mg/kg (Shacklette and Boerngen 1984), and Mo has been shown to have quite low toxicity to terrestrial plants, soil invertebrates, and critical soil processes (ACLs; Table2). Thus, background concentrations in most US soils are orders of magnitude less than the ACLs for Mo (Table2) that are protective of plants, soil invertebrates, and critical soil processes. Because Mo background concentrations are dramatically less than the ACLs for Mo, in the case of Mo the ACLs effectively become examples of resulting SCVs for site soils that correspond to the soil normalization characteristics specified in Table2. For example, the SCVs that result for coarse-textured site-specific soils that have pH = 6 and 10% clay content based on the 5th percentile from the SSD of EC50 values is 84 mg Mo/kg soil whereas selection of the 50th percentile from the SSD of EC10 values yields 328 mg Mo/kg soil. When coarse-textured site-specific soils have pH = 7 and 10% clay content, the SCVs based on regulators (e.g., USEPA) selecting the 5th percentile of the EC50 values becomes 21 mg Mo/kg soil, whereas selection of the 50th percentile of EC10 values yields 206 mg Mo/kg soil. These resulting SCV values comport with the experimental findings that the toxicity of the added Mo anion largely increases with increasing soil pH (McGrath et al. 2010).

After substantial review and evaluation, the USEPA focused Eco-SSL efforts for protection of plants and soil invertebrates on the utilization of EC20 data. This was done in large part because EC20 is a relatively low-level effect on groups of individuals or populations and has substantially less error and uncertainty associated with it than does lower-level ECx data (e.g., EC10). Should regulators promulgate the SCV estimation using an intermediate level effect (e.g., EC20) or an intermediate level of protection (e.g., HC25; 75% of species protected from an ECx population effect), the resulting SCV concentrations for Mo would be intermediate to the calculated examples provided (Table2).

## RESEARCH RECOMMENDATIONS

In both the REACH and Australian EIL databases, there are limitations in the number of effect concentrations data recorded, hence a compilation of databases is recommended to allow for derivation of SCVs. For less common metals and/or metalloids, and other chemicals, existing databases might not have complete sets of soil properties with associated effect concentrations at different levels of effects (i.e., ECx), thus additional investigation might be required.

The case study (Table2) illustrates that several choices can be made when deriving SCVs from the SSDs (i.e., different combinations of ECx and HCy). The question arises about which HCy level of protection is most appropriate for each type of ECx SSD: Is a higher number of species affected reasonable when the adverse effect per species is relatively small, or is it better to have a larger effect on fewer species? It is unlikely that field data may unequivocally yield the answer; therefore, policy and regulatory decision makers should clearly communicate their strategy and rationale to the general public and other stakeholders. However, site-specific field data can be used to assist with this decision making, and regulator decisions may be reinforced with different lines of evidence. Integration of separate lines of evidence will further reduce the uncertainty associated with SCVs derived for specific sites undergoing assessment.

The bioavailability corrections by normalization, recommended above, neither account for metal speciation nor the source of the site-specific contamination. For some metals or metal compounds, differences in solubility compared with that of metal salts are large. Soil extraction protocols that distinguish adsorbed metals from biologically unavailable metals, when such procedures become available, will be advantageous additions to the methods recommended herein. Using proven soil extraction procedures should reduce uncertainty in risk identification, might eliminate reliance solely on effects assessment from toxicity tests based on highly bioavailable metal, and may bridge the gap between metal-spiked and field-contaminated soils.

## SUPPLEMENTAL DATA

**Table S1.** Summary of steps involved in applying the proposed new method for deriving site-specific Soil Clean-up Values (SCVs) to protect the ecological receptors plants and soil invertebrates (may also include soil microbial processes, as in this Case Study).

**Figure S1.** Overview of Process for Derivation of a Final Soil Quality Guideline in Canada (CCME 2007; with permission).

**Figure S2.** Procedure for Deriving Soil Quality Guidelines for the Soil Contact Exposure Pathway (SQG_SC_) for Agricultural and Residential Land Use in Canada (CCME 2007; with permission).

**Supporting Information**: List of Abbreviations.

## Supporting Information

All Supplemental Data may be found in the online version of this article.

Table S1 Summary of steps involved in applying the proposed new method for deriving site-specific Soil Clean-up Values (SCVs) to protect the ecological receptors plants and soil invertebrates (may also include soil microbial processes, as in this Case Study).

Figure S1 Overview of Process for Derivation of a Final Soil Quality Guideline in Canada (CCME 2007; with permission).

Figure S2 Procedure for Deriving Soil Quality Guidelines for the Soil Contact Exposure Pathway (SQG_SC_) for Agricultural and Residential Land Use in Canada (CCME 2007; with permission).

Supporting Information :List of Abbreviations.
