# Supplementary material for: Deriving site-specific clean-up criteria to protect ecological receptors (plants and soil invertebrates) exposed to metal or metalloid soil contaminants via the direct contact exposure pathway
Source: Integr Environ Assess Manag. 2014 Jan 1;10(3):346–57. doi: 10.1002/ieam.1528 (PMC4285199; doi:10.1002/ieam.1528)
Supplement: Table S1 — Summary of steps involved in applying the proposed new method for deriving site-specific Soil Clean-up Values (SCVs) to protect the ecological receptors plants and soil invertebrates (may also include soil microbial processes, as in this Case Study). [file ieam0010-0346-SD1.doc]

Supplemental Table 1. Summary of steps involved in applying the proposed new method for deriving site-specific Soil Clean-up Values (SCVs) to protect the ecological receptors, plants and soil invertebrates (may also include soil microbial processes, as in this Case Study).

Following screening level ecological risk assessment (e.g., in the USA using Ecological Soil Screening Levels), the approach for a metal or metalloid in the context of baseline ecological risk assessment is:

1. Compile data that meet selection criteria established for derivation of Eco-SSLs, except include study results that have:

a) Soil pH or organic matter content (OM%) outside Eco-SSL criteria limits

b) Endpoints for microbial processes linked to soil fertility or other critical soil processes

1. Sort data into EC_x_ data sets; express data as “total added” concentration
2. Apply Leaching-Aging Factor (LAF) corrections
3. Normalize data sets to selected soil; based on best correlations between soil characteristic and toxicity measurement endpoint
4. Calculate geomean for each species tested, and assemble EC_x_ SDDs
5. Select HC_y_ value (percentile protection for EC_x_-based distributions); expressed as an added contaminant limit (ACL)
6. Normalize ACL to site-soil, then add to background concentration to obtain the site-specific SCV
