# Supplementary figures and images for: Deriving site-specific clean-up criteria to protect ecological receptors (plants and soil invertebrates) exposed to metal or metalloid soil contaminants via the direct contact exposure pathway
Source: Integr Environ Assess Manag. 2014 Jan 1;10(3):346–57. doi: 10.1002/ieam.1528 (PMC4285199; doi:10.1002/ieam.1528)

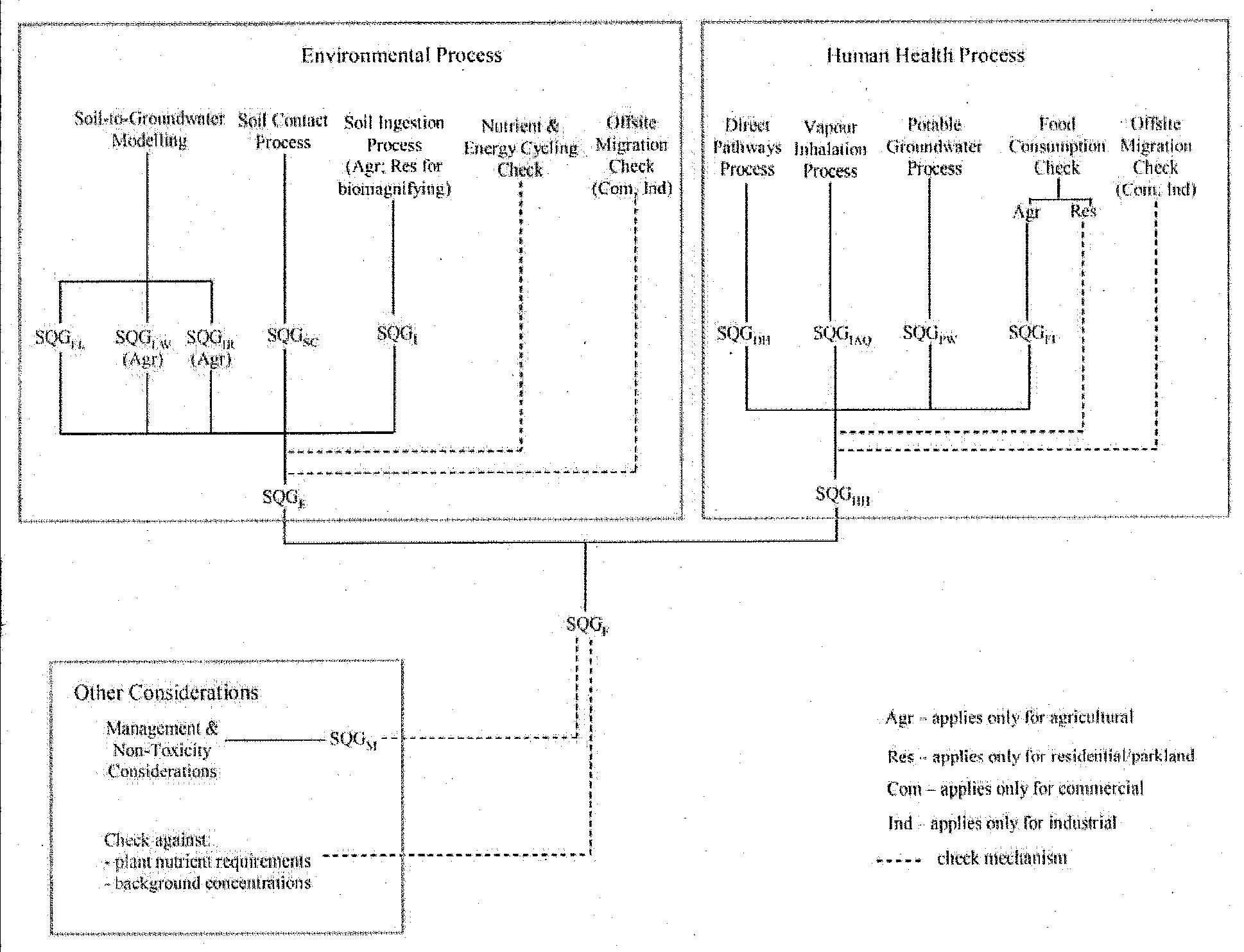

Supplement: Figure S1 — Overview of Process for Derivation of a Final Soil Quality Guideline in Canada (CCME 2007; with permission). [file ieam0010-0346-SD2.jpg]

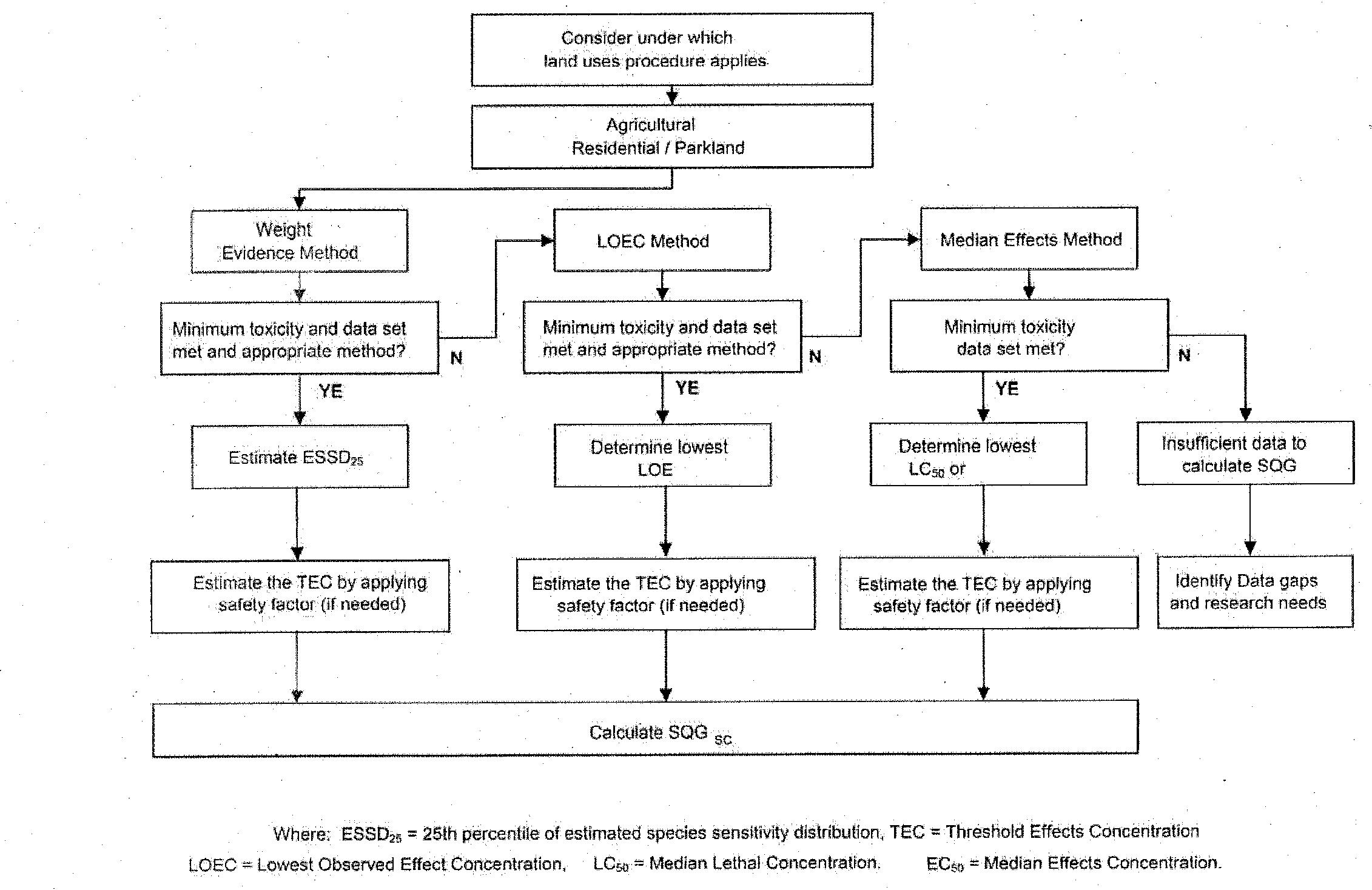

Supplement: Figure S2 — Procedure for Deriving Soil Quality Guidelines for the Soil Contact Exposure Pathway (SQGSC) for Agricultural and Residential Land Use in Canada (CCME 2007; with permission). [file ieam0010-0346-SD3.jpg]
