# Supplementary material for: Deriving site-specific clean-up criteria to protect ecological receptors (plants and soil invertebrates) exposed to metal or metalloid soil contaminants via the direct contact exposure pathway
Source: Integr Environ Assess Manag. 2014 Jan 1;10(3):346–57. doi: 10.1002/ieam.1528 (PMC4285199; doi:10.1002/ieam.1528)
Supplement: Supporting Information — :List of Abbreviations. [file ieam0010-0346-SD4.doc]

**SUPPLEMENTAL MATERIALS**

**List of Abbreviations**

ACL Added Contaminant Limit

AF Assessment Factor (also known as uncertainty factor, application factor, or safety

factor); an arbitrary value implemented in various ways to increase

conservatism in soil limit decisions

BC-MOE British Columbia Ministry of Environment (Canada)

BERA Baseline ecological risk assessment

CCME Canadian Council of Ministers of the Environment

CERCLA Comprehensive Environmental Response, Compensation, and Liability Act

EC Environmental Canada

EC_x_ Exposure concentration that yields a specified x-percentage effect (e.g., EC_50_)

ECB European Chemicals Bureau

eCEC Effective cation exchange capacity

ECHA European Chemicals Agency

ECL Effects concentration – Low

Eco-SSL Ecological Soil Screening Level

EIL Ecological Investigation Level; the sum of the ACL (Added Contaminant Limit)

and the corresponding ambient background concentration, for a specific

contaminant and site

ERA Ecological Risk Assessment

EU European Union

Geomean Geometric mean (n^th^ root of the product of 'n' numbers)

HC_y_ Hazardous concentration for y% of a species (the concentration that protects

‘100 – y’ percent of a species)

IC_x_ Inhibitory concentration; concentration that yields a specified x-percentage

inhibitory effect (e.g., IC_25_)

ISO International Organization for Standardization

LAF Leaching-Aging Factor

LC_x_ Concentration lethal to a specified x-percentage of test population (e.g., LC_50_)

LOAEC Lowest observed adverse effect concentration

LOEC Lowest observed effect concentration

MATC Maximum acceptable toxicant concentration

NEPA National Environmental Policy Act (USA)

NEPC National Environment Protection Council, Australia

NOAEC No observed adverse effect concentration

NOEC No observed effect concentration

NRCS Natural Resources Conservation Service; formerly Soil Conservation Service,

SCS (USA)

OECD Organisation for Economic Co-operation and Development

OM Organic matter

Ontario-MOE Ontario Ministry of Environment (Canada)

PNEC_soil_ Predicted No Effect Concentration in Soil (EU)

REACH Registration Evaluation Authorisation and Restriction of Chemicals;

EU Regulation

SCV Soil Clean-up Value

SLERA Screening level ecological risk assessment

SQG Soil quality guideline; SQG_E_ for Environment, SQG_F_ for Final, SQG_HH_ for

Human Health, SQG_SC_ for direct Soil Contact, SQG_(NOAEC & EC10)_ developed from NOAEC and EC_10_ data, SQG_(LOAEC & EC30)_ developed from LOAEC and EC_30_ data, SQG_(EC50)_ developed from EC_50_ data

SSD Species Sensitivity Distribution

t-BLM Terrestrial biotic ligand model

TEC Threshold effect concentration

UF Uncertainty factor (also known as assessment factor, application factor, or safety

factor); an arbitrary value implemented in various ways to increase

conservatism in soil limit decisions

USA United States of America

USEPA U.S. Environmental Protection Agency (USA)

USGS U.S. Geological Survey (USA)
